# A journey full of surprises: surgical insights from pediatric undifferentiated embryonal liver sarcomas

**DOI:** 10.1007/s00383-025-06101-y

**Published:** 2025-06-25

**Authors:** Senol Emre, Ayse Karagoz, Ali Ekber Hakalmaz, Suheyla Ocak, Ayse Kalyoncu Ucar, Nuray Kepil, Pinar Kendigelen, Cigdem Tutuncu, Sebuh Kurugoglu, Osman Faruk Senyuz

**Affiliations:** 1 Department of Pediatric Surgery, Istanbul University-Cerrahpasa, Cerrahpasa School of Medicine, Istanbul, Turkey; 2 Department of Pediatric Hematology and Oncology, Istanbul University-Cerrahpasa, Cerrahpasa School of Medicine, Istanbul, Turkey; 3 Department of Pediatric Radiology, Istanbul University-Cerrahpasa, Cerrahpasa School of Medicine, Istanbul, Turkey; 4 Department of Pathology, Istanbul University-Cerrahpasa, Cerrahpasa School of Medicine, Istanbul, Turkey; 5 Department of Anesthesiology and Reanimation, Istanbul University-Cerrahpasa, Cerrahpasa School of Medicine, Istanbul, Turkey

## Abstract

**Aim:**

Undifferentiated embryonal sarcoma of the liver (UESL) presents significant diagnostic and therapeutic challenges due to its rarity and unpredictable clinical course. This study emphasizes the importance of individualized, case-based assessment by highlighting the distinctive preoperative, intraoperative, and postoperative challenges encountered in pediatric UESL patients.

**Material and methods:**

We performed a retrospective review of nine pediatric patients treated for UESL at our institution from 2012 to 2022. We systematically evaluated clinical presentations, radiological assessments, surgical techniques, perioperative findings, treatment protocols, and follow-up outcomes.

**Results:**

Each patient presented unique diagnostic and therapeutic challenges at various management stages. Preoperatively, overlapping radiologic and immunohistochemical features led to diagnostic uncertainty; notably, weak β-catenin positivity and low AFP levels initially resulted in misdiagnosis and incorrect treatment protocol application in one case. Significant intraoperative events included unexpected pulmonary embolism, while another patient developed posterior reversible encephalopathy syndrome (PRES) immediately preoperatively, delaying surgical intervention. Excluding one patient who experienced an unexplained sudden death on postoperative day 5, no other major surgical complications (such as hemorrhage, infection, bile leakage, biliary complications, or sepsis) were recorded. At a median follow-up of 7.2 years (range: 13 months to 12 years), five patients remained disease-free, one patient continued with intermittent chemotherapy, two patients succumbed to progressive metastatic recurrence, and one patient died postoperatively on day 5 due to an unexplained event.

**Conclusion:**

UESL requires individualized, case-based evaluation and multidisciplinary collaboration. Each patient may present unique diagnostic, surgical, and postoperative management challenges. Awareness of potential complications and the unpredictable nature of UESL highlights the importance of centralized, multidisciplinary care to optimize clinical outcomes.

## Introduction

Undifferentiated embryonal sarcoma of the liver (UESL) is a rare and aggressive pediatric malignancy, 6–13% of malignant hepatic tumors in children [1–3]. Although significant progress has been made through multimodal therapy—including surgical resection and chemotherapy—UESL continues to present diagnostic and therapeutic challenges not fully addressed by existing protocols [4]; [5]. The limited number of cases worldwide has precluded the development of standardized management strategies that mirror those implemented for more common pediatric solid tumors. Consequently, clinicians may encounter difficulties during tumor characterization, perioperative planning, and postoperative surveillance. In particular, the absence of a reliable tumor marker complicates both early detection and long-term follow-up, and histopathological misinterpretation can lead to inappropriate or delayed treatment. While published literature supports the efficacy of neoadjuvant therapies—ranging from conventional chemotherapy to transarterial chemoembolization (TACE)—individual responses remain variable, sometimes resulting in progression rather than tumor shrinkage. Adding to the complexity, large post-resection regenerative nodules can mimic residual or recurrent disease on imaging, requiring advanced modalities such as diffusion-weighted MRI for more accurate differentiation [4].

In this report, we present our institution’s experience with 9 pediatric UESL cases, highlighting the multifaceted challenges at each step of management—from initial biopsy and radiologic assessment to operative technique and postoperative follow-up. We aim to illustrate the unpredictable nature of UESL while emphasizing the need for a rigorous, case-by-case approach. We also discuss the value of centralizing care to build expertise, refine institutional protocols, and ultimately improve outcomes in this rare pediatric entity.

## Materials and methods

A retrospective review was conducted of all patients diagnosed with Undifferentiated Embryonal Sarcoma of the Liver (UESL) and treated at our clinic between 2012 and 2022. Recorded data included patient age, sex, presenting symptoms, tumor markers, comorbidities, radiological diagnostic methods, histopathology reports, surgical procedures, chemotherapy and radiotherapy treatments, follow-up information, and anecdotal clinical observations noted during the treatment process. Due to the retrospective and anonymized nature of this study, formal ethics approval was waived by our institution’s ethics committee. Informed consent for treatment and publication was obtained from the parents or legal guardians of all included patients.

Following admission, a standardized approach was applied to each case. At the initial evaluation of patients with a suspected liver mass, serum alpha-fetoprotein (AFP) levels, general biochemical analyses, and abdominal ultrasounds were performed. For further characterization of the lesion, evaluation of additional lesions, and screening for cranial metastases, intravenous (IV) contrast-enhanced magnetic resonance imaging (MRI) was used. IV contrast-enhanced computed tomography (CT) was performed to assess vascular involvement and detect potential bone and lung metastases. Positron emission tomography (PET) was also employed in selected patients.

In cases where the lesion displayed a predominantly solid component and where radiological findings suggested a differential diagnosis other than mesenchymal hamartoma, fine-needle aspiration biopsy (FNAB) and Tru-Cut biopsy were performed. Parenchymal access was prioritized to minimize the risk of bleeding and rupture. In patients strongly suspected of having mesenchymal hamartoma, surgery was undertaken without a prior biopsy.

Decisions regarding neoadjuvant therapy for UESL were made on a case-by-case basis by the Pediatric Oncology Council. During surgical resection, lymph nodes in the hepatic pedicle were excised to achieve an R0 resection. A minimum surgical margin of 2 cm was targeted; thus, extended resections were preferred if the expected remnant liver volume was deemed sufficient.

## Results

Between 2012 and 2022, we treated nine pediatric UESL patients (4 female, 5 male; ages 3–17 years, mean 8.4). Seven presented with palpable abdominal mass, one with weight loss/jaundice, and one with an incidental lesion. All tumors were solitary. PRETEXT staging showed five patients with right lobe tumors (PRETEXT II), two with right lobe and left medial sector involvement (PRETEXT III), one with left lobe/right anterior sector involvement (PRETEXT III), and one with central segment involvement extending to segment 2 (PRETEXT III). Portal vein involvement occurred in two patients and IVC involvement in one. Tumor size ranged from 10-22 cm (mean 14.3 cm). Two patients had liver metastases at diagnosis. Radiologically, six cases showed characteristic UESL features, while two mimicked mesenchymal hamartoma and one resembled hepatoblastoma. Diagnosis was established via percutaneous biopsy in seven cases and from resection specimens in two. Histopathology showed desmin and vimentin positivity in all cases. One three-year-old with beta-catenin positivity was initially misdiagnosed as anaplastic hepatoblastoma, with UESL confirmed after resection. Five patients received neoadjuvant Ifosfamide-Adriamycin chemotherapy, one received SIOPEL 4 protocol (initially suspected hepatoblastoma), and three received no neoadjuvant therapy. Among neoadjuvant-treated cases, three showed no radiological regression, and two showed tumor enlargement. We achieved R0 resection in seven cases. One case with extensive IVC tumor thrombus and proper hepatic artery involvement underwent R1 resection with adventitiectomy. Another case had R2 resection with devascularization and intratumoral alcohol injection.

No surgical complications occurred. All patients received the adjuvant VAC regimen (vincristine, actinomycin-D, cyclophosphamide). One recurrence received whole-abdominal radiotherapy. A summary of every patient’s diagnostic and therapeutic journey is presented in Table 1.

**Table 1:** Summary of the cases

Case	Age and Sex	Mass size and localization	AFP	Metastasis	PRETEXT	Biopsy	Neoadjuvant Regimen	Radiological Response	POSTTEXT	Pathological Response	Surgery	Outcome	Follow-up period
1	10, F	**13 cm** Right Lobe	Normal	None	II	FNAB	None	N/A	N/A	N/A	R0 Extended hepatectomy	NED	12 yrs
2	7, F	**16 cm** Right Lobe	Normal	None	II	Tru-cut (2 times- non-diagnostic)	None	N/A	N/A	Non-diagnostic initial biopsy	R0 Extended hepatectomy	NED	6 yrs
3	3, M	**16 cm** Right Lobe	Normal	None	II	Tru-cut	SIOPEL-4 (2 cycles)	No response	II	Not standardized*	R0 Left extended hepatectomy	NED	10 yrs
4	8, M	**12 cm** Left Lobe and Right Anterior Sector-PV-Cavernous Malformation	Normal	Yes, PV	III + P	Tru-cut	Ifosfamide-Adriamycin	Progressive disease	III + P	Necrosis (5%) minimal hyalinization	R2 Devascularization	Stable disease	10 yrs
5	4, M	**10 cm** Segments 2, 4, 5 and 8	Normal	None	III	N/A Intraoperative wedge bx	None	N/A	III	N/A	R0 Left extended hepatectomy	DOD	2 yrs
6	9, M	**18 cm** Right Lobe	Normal	Lung	II	FNAB	Ifosfamide-Adriamycin (4 cycles)	No response	II	Necrosis 30–40%, extensive fibrosis/hyalinization**	R0 Right extended hepatectomy	Died perioperatively	12 yrs
7	10, F	**14 cm** Right Lobe	Normal	none	II	Tru-cut	None	N/A	N/A	N/A	R0 Right hepatectomy	NED	9 yrs
8	8, M	**12 cm** Right Lobe + Left medial sector	Normal	None	III	Tru-cut	Ifosfamide-Adriamycin	No response	III	Not standardized*	R0 Segmentectomy	NED	3 yrs
9	17, F	**22 cm** Right Lobe + Left medial sector-IVC-İliac Veins	Normal	Yes-IVC,, Lung	III + IVC	Tru-cut-FNAB	Ifosfamide-Adriamycin	Progressive disease	III + IVC + P	15–20% treatment effect (13% fibrosis, 2% necrosis, 85% viable tumor)	R1 Extended hepatectomy	DOD	13 months

* Standardized pathological response assessment criteria not available

** Secondary treatment changes: Extensive hyalinization, fibrosis findings

All patients received adjuvant VAC (Vincristine, Actinomycin-D, Cyclophosphamide).

*NED* No evidence of disease, *DOD* Died of disease, *R0* Complete resection with negative margins, *R1* Microscopic positive margins, *R2* Macroscopic residual disease.

### Anecdotal key points and case events

Case 1: Postoperatively, 4–5 regeneration nodules developed (largest 1 cm), difficult to identify on ultrasound. The absence of a follow-up marker and poor nodule visualization challenged recurrence monitoring. A patient-specific follow-up protocol was eventually developed.

Case 2: Initial trucut biopsy was non-diagnostic with insufficient healthy liver tissue and inconclusive desmin/vimentin staining. The tumor grew from 12 to 16 cm during the one-month biopsy period. Preoperative imaging (Doppler ultrasound, IV contrast CT, echocardiography) showed no vascular invasion. During exploration with Mercedes incision, a sudden drop in end-tidal CO2 during liver mobilization was followed by cardiac arrest. Sternotomy for internal cardiac massage was performed, and intraoperative echocardiography revealed a 1 × 5 cm tumor embolism in the right pulmonary artery. Left pulmonary embolectomy under total circulatory arrest was performed. Despite heparinization risks, total extended hepatectomy with total vascular isolation proceeded. The patient required ECMO and ventilatory support until postoperative day 9 after 75 min of internal cardiac massage but was extubated without neurological or physical sequelae. Currently has an asymptomatic right-sided diaphragmatic hernia under observation due to family concerns about further surgery.

Case 3: Initial biopsy showed weak vimentin positivity, positive beta-keratin, mitotic activity, and hyperchromatic nuclei. Despite negative AFP, anaplastic hepatoblastoma was considered in this 3-year-old, and SIOPEL high-risk protocol initiated. Treatment was discontinued after two cycles due to a lack of size response. Resection revealed UESL, and treatment was adjusted accordingly.

Case 4: Tumor occupied the left lobe extending to Segment 8, encompassing the middle hepatic vein and abutting right/left hepatic veins. Portal vein thrombosis and cavernous malformation were present. Despite a visible hepatic venous return on CT and the absence of Budd-Chiari syndrome, the patient (blood type O Rh-) had no suitable living donor. Marginal resection was attempted but abandoned intraoperatively. Management included left hepatic artery/portal vein ligation, portal vein thrombectomy, and intratumoral 97% alcohol injection. Postoperatively, except for two days of high fever, no complications occurred. By the third month, the tumor size decreased by 5 cm with cystic changes and increased capsular echogenicity. Biopsies revealed fibrotic-hyalinized portal vein "thrombosis" (not tumor thrombus) and treatment response within the tumor. The patient has maintained stable disease with intermittent chemotherapy for 10 years.

Case 5: A 4-year-old with abdominal pain and fever had a 10 cm mass involving segments 2, 4, 5, and 8. MRI/CT showed a lobulated, heterogeneous mass with solid, cystic, and necrotic components, apparently involving the gallbladder and vascular structures. Mesenchymal hamartoma was suspected; surgery was planned without biopsy. Despite normal preoperative evaluations, the patient developed sudden vision loss on the morning of surgery. Cranial MRI revealed T2/FLAIR hyperintensities, diagnosed as PRES (Posterior Reversible Encephalopathy Syndrome). Surgery was postponed until symptoms were resolved (12 days). Intraoperative appearance prompted biopsy; frozen section showed slipper-shaped giant cells indicating malignancy but inconclusive lineage. Left extended hepatectomy was performed; histopathology confirmed UESL. After six adjuvant chemotherapy cycles, extensive liver/peritoneal metastases and two lung metastases developed at 20 months. The patient died in the second postoperative year from a progressive, chemotherapy-resistant disease.

Case 6: Despite lung metastasis, tumor size remained stable after neoadjuvant chemotherapy. With no radiological intravascular spread, right extended hepatectomy was performed without complications. Despite initial recovery (extubation, spontaneous respiration, oral feeding, normal blood pressure, serous drainage), the patient suffered sudden cardiac arrest on postoperative day 5. Pulmonary embolism from an undetected residual intravascular thrombus was suspected but unconfirmed as the family declined an autopsy.

Case 7: This 10-year-old female presented with a 14 cm right lobe tumor and represents one of our cases with an unexpectedly straightforward clinical course. Despite the absence of neoadjuvant therapy, intraoperative findings showed a well-encapsulated tumor with clear anatomical planes, allowing for an uncomplicated extended right hepatectomy with R0 resection. The patient's postoperative recovery was unremarkable, and he remains disease-free at 9 years follow-up.

Case 8: An 8-year-old male with a 12 cm tumor who underwent neoadjuvant chemotherapy without radiological response. Despite the lack of tumor shrinkage, which initially raised concerns about potential surgical complications and tumor friability, the intraoperative findings were surprisingly favorable. The tumor demonstrated clear surgical planes and adequate capsular integrity, enabling successful R0 resection without technical difficulties.

Case 9: A 17-year-old female presented with right upper quadrant mass causing rib cage deformation, 10 kg weight loss, jaundice, and caput medusae. Imaging showed an 18 cm mass involving the right lobe/left medial sector with caudal extension. IVC contained tumor thrombus from the iliac veins to the suprahepatic IVC. The right hepatic artery was circumferentially encased (360°), proper hepatic artery was involved (270°). Bile duct compression caused intrahepatic duct dilation. Two 7 mm right lower lobe lung metastases were present. Pre-neoadjuvant management included PTC biliary decompression and prophylactic suprahepatic IVC filter placement. Despite Ifosfamide-Adriamycin chemotherapy, the tumor enlarged. Deemed unsuitable for transplantation, the patient underwent right extended hepatectomy, portal vein thrombectomy, en bloc adventitial resection around the proper hepatic artery, and removal of IVC tumor thrombus extending to the femoral veins with venorrhaphy. The initial response to adjuvant VDC chemotherapy was good, but a chemo-radioresistant porta hepatis mass developed at 11 months, leading to death at 13 months post-surgery.

Excluding the patient who experienced an unexplained sudden death on postoperative day 5, no surgery-related postoperative complications—such as small-for-size syndrome, hemorrhage, bile leakage, biliary tract issues, infection, or other complications—were observed in our series.

At an average follow-up of 7.2 years (range: 13 months to 12 years), of the nine cases reviewed, five patients are alive without complications while one remains on intermittent chemotherapy. Two patients succumbed to recurrence with extrahepatic metastasis, and one patient died on postoperative day 5.

## Discussion

Undifferentiated embryonal sarcoma of the liver (UESL) is a rare pediatric malignancy [2]. Due to its low incidence, it remains difficult to accumulate substantial institutional experience, and standardized treatment protocols are limited. While the published literature often reports favorable outcomes with multimodal therapy, individual UESL cases can present unexpected diagnostic or therapeutic challenges that may not be fully captured in larger series. Indeed, the emphasis on aggregate, standardized outcomes in cohort reports can inadvertently mask case-specific complexities. Therefore, this manuscript provides a detailed, case-based exploration of these anecdotal presentations and complications to illustrate the nuanced realities of UESL management.

Adequate tissue sampling critically determines diagnostic accuracy in UESL, yet procedural complexities often compromise specimen acquisition. While core needle biopsy (Tru-Cut) represents the gold standard, UESL's highly vascular nature leads inexperienced interventional radiologists to default to fine-needle aspiration biopsy (FNAB), minimizing bleeding risk but often yielding insufficient material for definitive characterization. Case 2 exemplifies this challenge, where initial FNAB failure necessitated repeat sampling, causing diagnostic delay and tumor progression. Optimal technique requires traversing healthy hepatic parenchyma rather than directly accessing tumor capsule, reducing rupture risk and tumor seeding. Concurrent sampling of adjacent normal liver tissue provides a comparative histological context and enables assessment of chemotherapy-induced hepatotoxicity, particularly veno-occlusive disease (VOD). Our current protocol mandates direct collaboration between pediatric surgeons and interventional radiologists during biopsy procedures, significantly improving specimen adequacy while maintaining safety in rare pediatric hepatic malignancies.

Pathological response evaluation in our series was limited by the inherent challenges of retrospective data collection spanning a decade-long period. While neoadjuvant chemotherapy demonstrated minimal radiological efficacy with no cases achieving significant tumor shrinkage, comprehensive pathological response assessment was complicated by evolving institutional reporting standards and variable documentation practices across the study timeframe. Available histopathological data from resected specimens suggested heterogeneous treatment effects, with some cases demonstrating focal areas of therapy-induced necrosis, fibrosis, and hyalinization, though substantial viable tumor burden persisted in most instances. The inconsistency in pathological response reporting—ranging from detailed quantitative assessments to descriptive observations—reflects the absence of standardized response evaluation criteria for UESL, a limitation that extends beyond our institution to the broader pediatric hepatic malignancy literature.

Given the poor correlation between neoadjuvant therapy and both radiological and pathological response in our experience, combined with successful R0 resection rates achieved regardless of preoperative treatment status, our current approach favors upfront surgical resection in technically resectable cases. This strategy minimizes treatment delays while avoiding potential chemotherapy-related complications in a disease where early complete resection remains the most critical prognostic factor.

UESL misinterpretation as anaplastic hepatoblastoma can arise from overlapping immunohistochemical markers and low alpha-fetoprotein (AFP) levels [6, 7]. In Case 3, extremely low AFP (< 100) combined with weak β-catenin positivity initially prompted high-risk hepatoblastoma therapy following SIOPEL guidelines. When the tumor failed to respond, repeat histopathological evaluation confirmed UESL. Although literature lacks definitive evidence of β-catenin expression in UESL, weak positivity in younger patients with normal/low AFP complicates diagnosis. For similarly ambiguous cases—especially PRETEXT II—considering Children's Oncology Group (COG) protocols may avoid unnecessary aggressive treatment and facilitate rapid UESL confirmation in complex pediatric liver tumors.

Although the literature offers promising examples of neoadjuvant therapy for UESL [8–12], our clinical experience has been less favorable, as patients receiving preoperative chemotherapy either progressed or showed no response. Ideally, neoadjuvant treatment should shrink and solidify the tumor to reduce the risk of rupture or pulmonary embolization—an event we encountered in a patient who did not receive such therapy. Conversely, two progressive tumors proved extremely challenging at surgery. In contrast, a recent Chinese series reported complete (R0) resection in all patients following neoadjuvant transarterial chemoembolization (TACE) plus systemic chemotherapy, achieving an average tumor volume reduction of 36% (range: 18–69%). These findings suggest TACE may be particularly useful for large or borderline-resectable UESLs by promoting both tumor shrinkage and intratumoral necrosis, thereby potentially mitigating residual microscopic disease [13]

Despite routine preoperative vascular assessment including contrast-enhanced CT and Doppler ultrasonography within 24 h of surgery, thromboembolic events remained unpredictable. Case 2 demonstrated clear vessels on immediate preoperative imaging yet developed confirmed intraoperative tumor embolism during liver mobilization, requiring emergency pulmonary embolectomy under total circulatory arrest. Case 6, despite neoadjuvant chemotherapy and no radiological vascular involvement, suffered a fatal suspected pulmonary embolism on postoperative day 5 following initially uncomplicated recovery—autopsy was declined, precluding definitive confirmation.

The mechanism likely involves spontaneous tumor fragmentation during surgical manipulation or hemodynamic changes, even when vessels appear radiologically clear. While neoadjuvant chemotherapy theoretically reduces embolization risk through tumor fibrosis, this protective effect was inconsistent: Case 2 underwent direct surgery and developed a confirmed embolism, while Case 6 received chemotherapy yet experienced suspected fatal embolism. Current imaging cannot reliably predict microscopic fragments or small intravascular extensions posing embolic risk during hepatic mobilization.

These experiences underscore the importance of establishing secure vascular control when imaging may underestimate tumor thrombi. When hepatic vein or IVC extension is suspected but not definitively visualized, prudent strategy involves securing vascular control before liver mobilization. Options include Rummel tourniquet around suprahepatic IVC for total vascular isolation or diaphragmatic incision to loop cavoatrial junction directly. Such precautionary maneuvers prevent sudden tumor embolization during manipulation, especially when imaging underestimates intravascular involvement. Preoperative communication with cardiovascular surgeons and intraoperative transesophageal echocardiography (TEE) facilitate early identification and management of embolic events, potentially reducing mortality and morbidity [14, 15].

PRES is a rare but significant complication in pediatric oncology patients, including those with hepatic tumors. It is primarily associated with hypertension, cytotoxic therapies, and major surgical interventions, all compromising cerebral autoregulation [16, 17]. While alarming, PRES is generally reversible with appropriate management, as demonstrated in our patient (Case 5), who achieved full recovery within 12 days. To our knowledge, PRES directly associated with hepatic malignancies such as UESL has not been previously reported. Importantly, PRES should not preclude continuing oncologic treatment; with prompt recognition and management, patients can safely resume therapy following symptom resolution.

Regenerative nodules following liver resection arise from compensatory hyperplasia driven by altered hemodynamics and proliferative signaling [18, 19]. In our UESL patient (Case 1), distinguishing benign from malignant nodules presented significant postoperative management challenges. While MRI demonstrates benign nodule characteristics (stable size, smooth contours, absence of diffusion restriction), routine MRI may not be feasible. Therefore, we recommend ultrasonography as primary imaging, reserving advanced techniques for suspicious features. Given the absent of specific biomarkers like AFP in UESL, clinicians should maintain caution and rely on multidisciplinary evaluation and careful follow-up, acknowledging ultrasonographic limitations while minimizing unnecessary interventions.

While the limited patient number and retrospective nature of this study may appear as methodological weaknesses, these characteristics paradoxically represent unique strengths when addressing rare pediatric malignancies like UESL. Retrospective case series serve a fundamentally different purpose than standardized multicenter trials—they capture the nuanced clinical reality that defines rare disease management. Each UESL case represents a unique educational opportunity, revealing diagnostic dilemmas, therapeutic challenges, and unexpected complications that cannot be adequately conveyed through standardized metrics alone.

The inherent variability in UESL presentation—from straightforward cases requiring minimal intervention to complex scenarios demanding innovative solutions—reflects the clinical reality that practicing surgeons must navigate. Rather than viewing this heterogeneity as a limitation to be minimized, we propose that it represents the core educational value of the case series. The anecdotal details—unexpected intraoperative events, diagnostic uncertainties, treatment failures—provide tactical knowledge essential for future clinical decision-making. This case-specific wisdom, accumulated through institutional experience, offers insights that standardized protocols cannot provide, particularly in diseases where textbook presentations are the exception rather than the rule.

Literature reports pediatric 5-year overall survival rates of 70–86% with complete surgical resection and multimodal therapy [5, 8, 20], significantly superior to adult outcomes (48.2%) [21, 22]. In our cohort, with a median follow-up of 7.2 years, event-free survival was 55.5% and overall survival was 66.6%. While these outcomes appear modestly lower than reported pediatric series, they reflect the challenges inherent in managing complex UESL cases, including advanced presentations and unexpected complications that may not be fully captured in aggregate literature reviews.

In conclusion, UESL remains unpredictable at every phase, often presenting unexpected diagnostic and therapeutic challenges. Its rarity hinders the establishment of robust protocols comparable to those for more common solid tumors, necessitating a highly individualized, case-based approach. Nevertheless, codifying stepwise management—from preoperative planning to perioperative decision-making and postoperative follow-up—can help mitigate the surprises inherent in UESL. Centralizing care in specialized centers is essential for building institutional expertise, refining protocols, and improving outcomes, especially as newer therapies such as TACE and advances in chemotherapy may convert otherwise inoperable or high-risk cases into viable surgical candidates.

## Data Availability

No datasets were generated or analysed during the current study.

## References

[1] Murawski M, Scheer M, Leuschner I et al (2020) Undifferentiated sarcoma of the liver: multicenter international experience of the cooperative soft-tissue sarcoma group and polish paediatric solid tumor Group. Pediatr Blood Cancer. 10.1002/PBC.28598 32706511 10.1002/pbc.28598

[2] Ismail H, Dembowska-Bagińska B, Broniszczak D et al (2013) Treatment of undifferentiated embryonal sarcoma of the liver in children - Single center experience. J Pediatr Surg 48:2202–2206. 10.1016/j.jpedsurg.2013.05.020 24210186 10.1016/j.jpedsurg.2013.05.020

[3] Kim DY, Kim KH, Jung SE et al (2002) Undifferentiated (embryonal) sarcoma of the liver: combination treatment by surgery and chemotherapy. J Pediatr Surg 37:1419–1423. 10.1053/jpsu.2002.35404 12378446 10.1053/jpsu.2002.35404

[4] Lin WY, Wu KH, Chen CY et al (2024) Treatment of undifferentiated embryonal sarcoma of the liver in children. Cancers (Basel). 10.3390/CANCERS16050897 38473259 10.3390/cancers16050897 PMC10931367

[5] Wu Z, Wei Y, Cai Z et al (2020) Long-term survival outcomes of undifferentiated embryonal sarcoma of the liver: a pooled analysis of 308 patients. ANZ J Surg 90:1615–1620. 10.1111/ANS.15684 31957153 10.1111/ans.15684

[6] Li Y, Cai Q, Jia N et al (2015) Pre-operatively misdiagnosed undifferentiated embryonal sarcoma of the liver: analysis of 16 cases. Ann Transl Med. 10.3978/J.ISSN.2305-5839.2015.11.20 26807408 10.3978/j.issn.2305-5839.2015.11.20 PMC4701533

[7] Rajaraman GB, Chandramouleeswari K, Regis MD et al (2024) Undifferentiated embryonal sarcoma of liver-a rare case entity. Nat J Lab Med. 10.7860/NJLM/2024/69371.2859

[8] Kastenberg ZJ, Short SS, Riehle KJ et al (2024) Management of undifferentiated embryonal sarcoma of the liver: a pediatric surgical oncology research collaborative study. Pediatr Blood Cancer. 10.1002/PBC.30975 38556718 10.1002/pbc.30975 PMC11039358

[9] Li J, He L, Wu Q et al (2024) Undifferentiated embryonal sarcoma of the liver in pediatric patients: a single-institution retrospective case series. Transl Pediatr. 10.21037/TP-24-10 38840683 10.21037/tp-24-10 PMC11148738

[10] Spunt SL, Xue W, Gao Z et al (2024) Embryonal sarcoma of the liver in pediatric and young adult patients: a report from Children’s Oncology Group study ARST0332. Cancer 130:2683–2693. 10.1002/CNCR.35305 38567652 10.1002/cncr.35305 PMC11260243

[11] Techavichit P, Masand PM, Himes RW et al (2016) Undifferentiated Embryonal Sarcoma of the Liver (UESL): a single-center experience and review of the literature. J Pediatr Hematol Oncol 38:261–268. 10.1097/MPH.0000000000000529 26925712 10.1097/MPH.0000000000000529

[12] Baron PW, Majlessipour F, Bedros AA et al (2007) Undifferentiated embryonal sarcoma of the liver successfully treated with chemotherapy and liver resection. J Gastrointest Surg 11:73–75. 10.1007/S11605-006-0044-4 17390190 10.1007/s11605-006-0044-4

[13] He M, Cai J-B, Lai C et al (2022) Neoadjuvant transcatheter arterial chemoembolization and systemic chemotherapy for the treatment of undifferentiated embryonal sarcoma of the liver in children. World J Clin Cases. 10.12998/wjcc.v10.i19.6437 35979288 10.12998/wjcc.v10.i19.6437 PMC9294901

[14] Fukuda K, Yoshimi A, Toma M et al (2021) Multi-disciplinary treatment of undifferentiated embryonal sarcoma of the liver with tumor embolus extending to the heart. J Pediatr Surg Case Rep. 10.1016/J.EPSC.2020.101705

[15] Salik I, Lamper N, Mehta B et al (2021) Intraoperative transesophageal echocardiography to monitor for pulmonary emboli in a pediatric patient undergoing undifferentiated embryonal sarcoma of the liver resection. Case Rep Anesthesiol. 10.1155/2021/5532028 34239733 10.1155/2021/5532028 PMC8238612

[16] Fugate JE, Rabinstein AA (2015) The diagnosis of posterior reversible encephalopathy syndrome - Authors’ reply. Lancet Neurol 14:1075. 10.1016/S1474-4422(15)00257-4 26466778 10.1016/S1474-4422(15)00257-4

[17] Lee VH, Wijdicks EFM, Manno EM et al (2008) Clinical spectrum of reversible posterior leukoencephalopathy syndrome. Arch Neurol 65:205–210. 10.1001/ARCHNEUROL.2007.46 18268188 10.1001/archneurol.2007.46

[18] Michalopoulos GK (2013) Principles of liver regeneration and growth homeostasis. Compr Physiol 3:485–513. 10.1002/CPHY.C120014 23720294 10.1002/cphy.c120014

[19] Fausto N, Campbell JS, Riehle KJ (2006) Liver regeneration. Hepatology. 10.1002/HEP.20969 16447274 10.1002/hep.20969

[20] Shi Y, Rojas Y, Zhang W et al (2017) Characteristics and outcomes in children with undifferentiated embryonal sarcoma of the liver: a report from the National Cancer Database. Pediatr Blood Cancer. 10.1002/PBC.26272 27781381 10.1002/pbc.26272 PMC5333454

[21] Ziogas IA, Zamora IJ, Lovvorn HN et al (2021) Undifferentiated embryonal sarcoma of the liver in children versus adults: a national cancer database analysis. Cancers (Basel). 10.3390/CANCERS13122918 34208030 10.3390/cancers13122918 PMC8230649

[22] Luong TTV, Mitchell C, Lokan J et al (2024) Long-term survival in an adolescent and young adult with metastatic relapse of an undifferentiated embryonal sarcoma of the liver. J Adolesc Young Adult Oncol 13:714–719. 10.1089/JAYAO.2023.0105 38579156 10.1089/jayao.2023.0105

